# Editorial—Special Issue: Foreword to the Special Issue on NIKE: Neuroendocrine Tumors, Innovation in Knowledge and Education

**DOI:** 10.3389/fendo.2021.722145

**Published:** 2021-07-01

**Authors:** Antongiulio Faggiano, Annamaria Colao

**Affiliations:** ^1^ Endocrinology Unit, Department of Clinical and Molecular Medicine, Sant’Andrea Hospital, Sapienza University of Rome, Rome, Italy; ^2^ Department of Clinical Medicine and Surgery, Federico II University of Naples, Unesco Chair Health Education and Sustainable Development, Naples, Italy

**Keywords:** Neuroendocrine neoplasm (NEN), Neuroendocrine tumors (NET), endocrine syndromes, paraneoplastic syndromes, advances therapies, molecular targets, efficacy, toxicity

Neuroendocrine neoplasms (NENs) are heterogeneous tumors arising from the diffuse neuroendocrine system, showing a common phenotype but variable behavior and prognosis. They include the well-differentiated neuroendocrine tumors (NETs), which are classically slow-growing and potentially functioning and the poorly differentiated neuroendocrine carcinomas (NECs), which are highly proliferative and aggressive (1, 2). NENs represent a model for the multidisciplinary management of tumors because different specialists are required, both for diagnosis and therapy. Not only oncologists and surgeons but also gastroenterologists, radiologists, nuclear medicine physicians and endocrinologists are needed for patients presenting with a wide spectrum of symptoms, either related to hormonal hypersecretion or tumor growth (3, 4). On the other hand, pathologists are indispensable to achieve the diagnosis and define the histo-prognostic category (5). Despite recent advances in the comprehension of biology and clinical behavior of these tumors, as well as the development of new diagnostic tools and therapeutic agents, many unmet needs remain to be addressed (6–8). Furthermore, quality of life and management of treatment toxicities represent a challenge in these patients who are characterized by long survival even at a metastatic stage (9–12).

This special issue is a mirror of the activities of the NIKE project on NET innovation in knowledge and education. This project started in 2015 with the aim of identifying the different clinical and biological unmet needs of NENs. This NIKE issue is a collection of four case reports, four retrospective studies, one original study and one perspective which describe unusual NEN primary sites and paraneoplastic syndromes, adverse events to NEN therapy, new therapeutic targets and strategies, prognostic factors and finally the minimum and optional requirements for a pathology report of NENs.

Secretory activity is one of the main characteristics of NENs. Despite nonfunctioning tumors are more frequent, several NEN-related endocrine and paraneoplastic syndromes can occur and need to be known and managed. Giannetta et al. reported four NET patients presenting with paraneoplastic hypercalcemia, due to PTH-related peptide in three cases and 1-25-dihydroxy-vitamin-D hypersecretion in one case. Paraneoplastic hypercalcemia is a rare condition that can be difficult to manage in NET patients. Zhang et al. reported a case of ectopic Cushing’s syndrome with unknown primary but presenting with lung lesions with a final diagnosis of nocardiosis. This is an opportunistic infection, usually related to immunosuppression, which represents a life-threatening condition of NET patients with ectopic Cushing’s syndrome and could be a confounding factor in the diagnostic work-up of NET.

The spectrum of therapeutic approaches for NEN has been enlarged in the last years. In parallel, treatment-related toxicities and cumulative toxicities need to be managed to ensure a good quality of life in these patients. Gubbi et al. reported a case of primary hypothyroidism following the first cycle of peptide receptor radionuclide therapy (PRRT) with ^177^Luthetium-DOTATATE for a metastatic paraganglioma. The intense thyroid uptake of ^177^Luthetium suggests a direct effect of this radionuclide through somatostatin receptors expressed in thyrocytes, with consequent marked increases in anti-Tg and anti-TPO antibodies and rapid development of primary hypothyroidism. Chang et al. underlines that everolimus, a mTOR inhibitor indicated in progressive NETs, can be associated with fulminant hepatitis. A patient presenting with a metastatic NET of the pancreas died three months after starting everolimus 10 mg a day, because of fulminant hepatitis due to reactivation of a chronic HBV infection. A specific prophylaxis is suggested in HBV positive patients undergoing therapy with everolimus. 

Despite NENs occurring mainly in gastroenteropancreatic and bronchial tracts, these tumors can show many other primary locations. Gallo et al. provide an accurate review of the main aspects of breast NENs, in order to better define an issue that has been subjected to subsequent changes in the classification criteria in the last years. Breast NENs are a rare subgroup of breast cancer but clear diagnostic criteria are still far from being established and the real size of this tumor could be underestimated.

Delving into the study of the mechanisms of NEN development, the fibroblast growth factor (FGF) pathway represents an intriguing oncogenic target. Vitale et al. analyzed its role in the development and progression of NENs, the occurrence of fibrotic complications and the onset of drug-resistance. Clinical trials with specific FGF-inhibitors are suggested to explore the role of the FGF pathway as molecular target for NEN therapy.

Di Molfetta et al. analyzed lights and shadows of immunotherapy in medullary thyroid cancer. This approach has been recently developed to induce an autoimmune response to the tumor and has found an excellent application in some cancer types like melanoma. In NEN, patient with merkelioma were found to be optimal candidates for immunotherapy, while less encouraging results have been observed in other NEN types. If available data on these new agents in medullary thyroid cancer are scarce at present, however some trials are now ongoing and a definitive conclusion on the role of immunotherapy in this setting will be addressed in the next years.

Lania et al. explore recent progress and future approaches of neoadjuvant therapy in NENs. This suggestive approach has been more and more relevant in many types of cancer, by using neoadjuvant chemo- or radiotherapy. In NEN there has been not a similar interest, likely for the peculiar biology and clinical course of these tumors which are, for the most part, slow growing, with low rates of proliferation and therefore less responsive to conventional anti-tumor therapies. A change in this view could be observed with PRRT, which is able to induce a significant rate of tumor shrinkage in NET expressing somatostatin receptors. Promising results are now available and other studies on this topic are ongoing.

An original study has been conducted by Barrea et al. on the role of metabolic syndrome and cardiometabolic indexes as prognostic factors in gastroenteropancreatic NETs. In the last years there is mounting evidence supporting the role of the metabolic syndrome in the pathogenesis of several tumors. In this study, the metabolic syndrome as well as the fatty liver index, a non-invasive tool for identifying individuals with non-alcoholic fatty liver disease, and the visceral adiposity index, a marker of adipose dysfunction, have been found to correlate with unfavorable clinicopathological characteristics.

Finally, Albertelli et al. provide a perspective article to analyze the main questions and relative answers, focusing on three main topics (i.e. morphology and classification, Ki67 and grading, immunohistochemistry), which were considered relevant for clinicians for understanding and correctly interpreting pathology reports on gastroenteropancreatic NENs. A minimum requirement in pathology report of NEN is also provided.

In summary, this special issue would be a support for endocrinologists, and for all other specialists involved in NEN management, by providing new insights in different fields of NEN, by suggesting new research lines and therapeutic targets and strategy, by reporting rare and unusual conditions related to NENs.

## Author Contributions

AF and AC both contributed to develop this article by resuming the results of all scientific manuscripts included in the Research Topic NIKE. All authors contributed to the article and approved the submitted version.

## Funding

This study was partially supported by the ministerial research project PRIN2017Z3N3YC.

## Conflict of Interest

The authors declare that the research was conducted in the absence of any commercial or financial relationships that could be construed as a potential conflict of interest.
